# Supplementary material for: Gut microbiome diversity is an independent predictor of survival in cervical cancer patients receiving chemoradiation
Source: Commun Biol. 2021 Feb 22;4:237. doi: 10.1038/s42003-021-01741-x (PMC7900251; doi:10.1038/s42003-021-01741-x)
Supplement: Supplementary file 2 — Supplementary Information [file 42003_2021_1741_MOESM2_ESM.pdf]

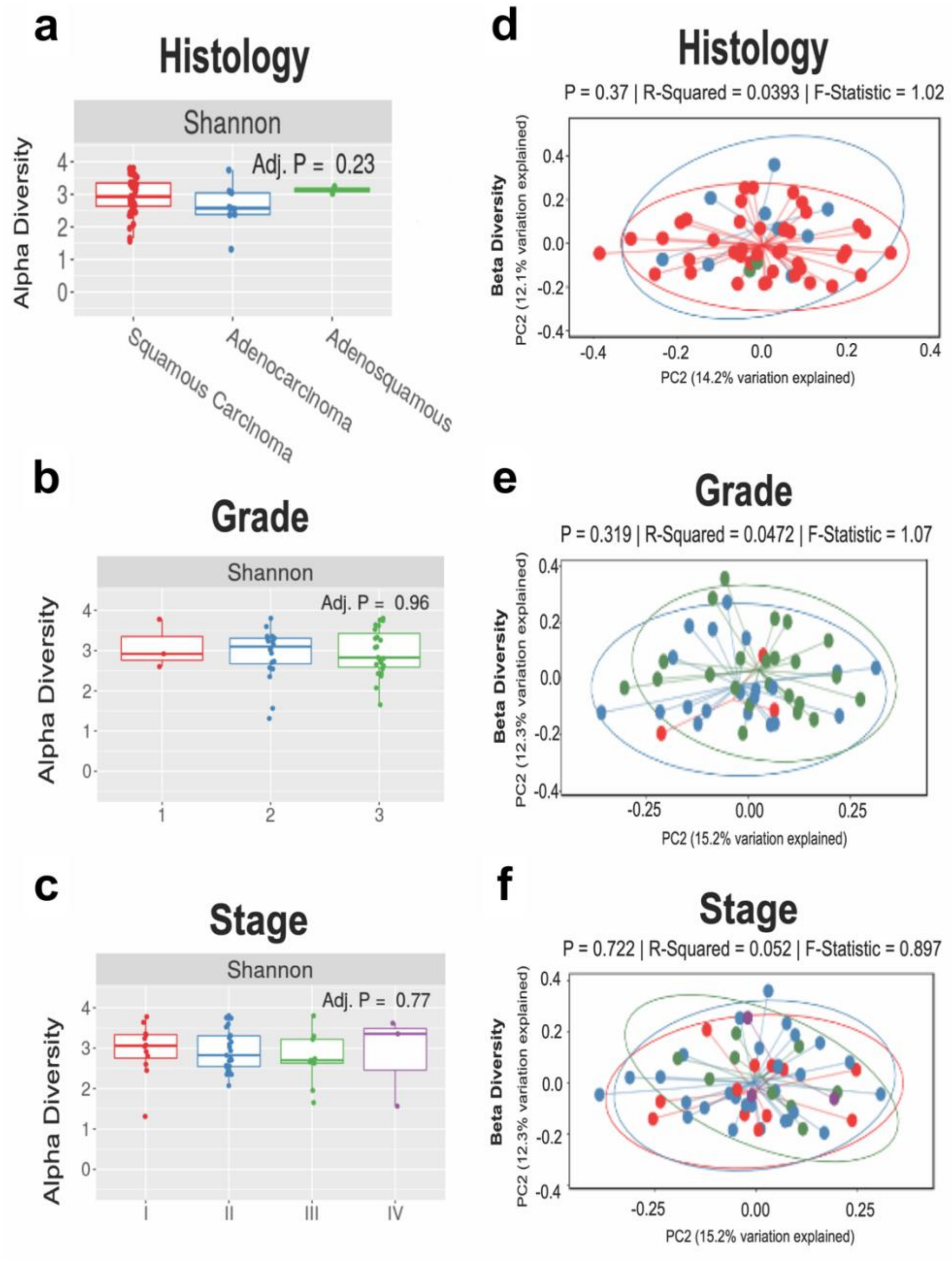

Supplemental Fig 1. The fecal microbiota of individuals with cervical cancer.

The fecal microbiota of individuals with cervical cancer by demographics. Alpha diversity (within sample diversity) was measured using the Shannon diversity metric and Beta diversity (between sample diversity) was determined by unweighted Unifrac. No differences were observed in either metric by cancer histology (A,D), grade (B,E) or cancer stage (C,F).

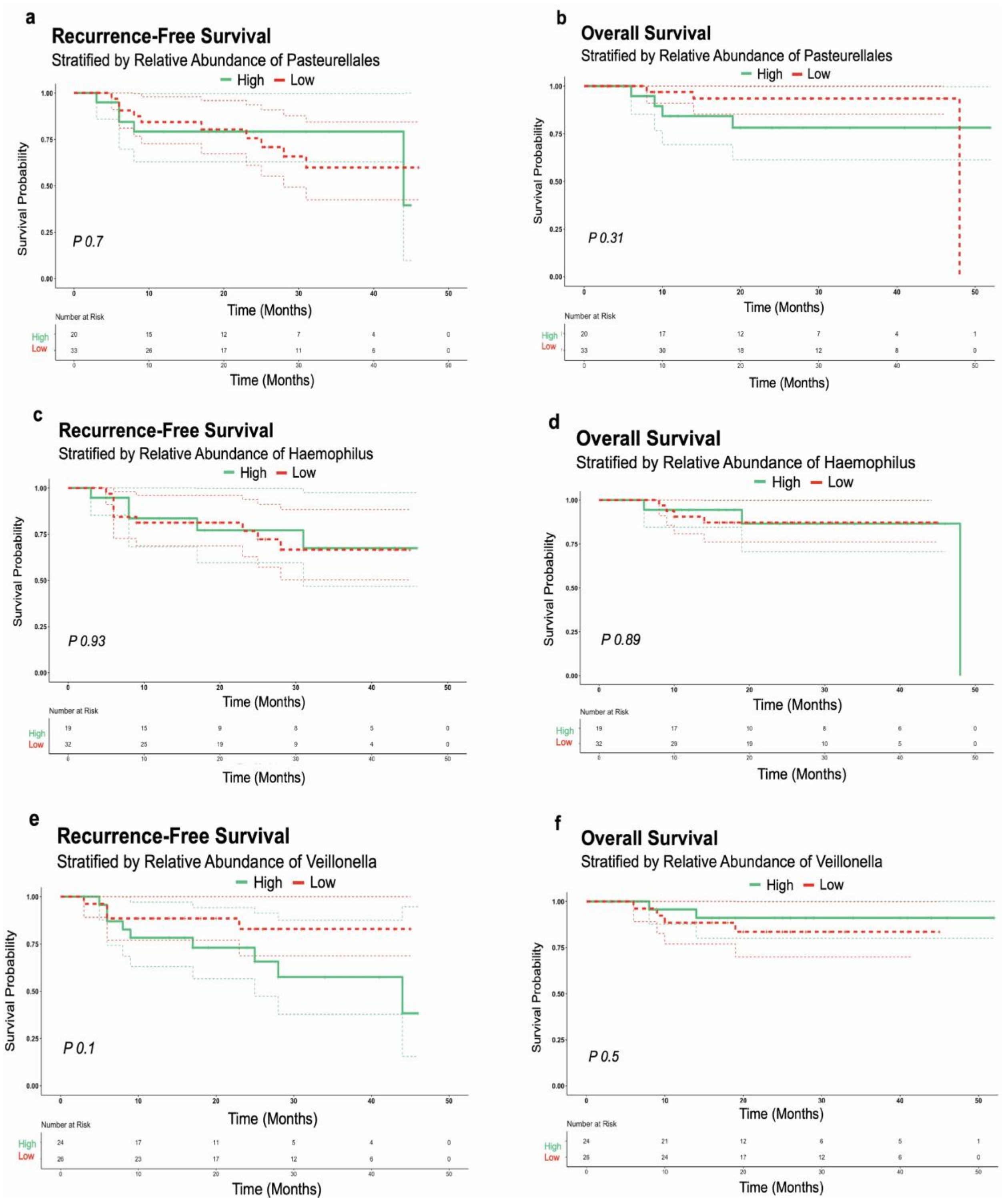

Supplemental Fig 2. Kaplan-Meier curves recurrence free and overall survival stratified by relative abundance of *Pasteurellales, Haemophilus, and Veillonella.*

Kaplan-Meier curves for (A) recurrence free survival, (B) overall survival stratified by relative abundance of *Pasteurellales*. Kaplan-Meier curves for (C) recurrence free survival, (D) overall survival stratified by relative abundance of *Haemophilus*. Kaplan-Meier curves for (E) recurrence free survival, (F) overall survival stratified by relative abundance of *Veillonella*. Cases represent patients.

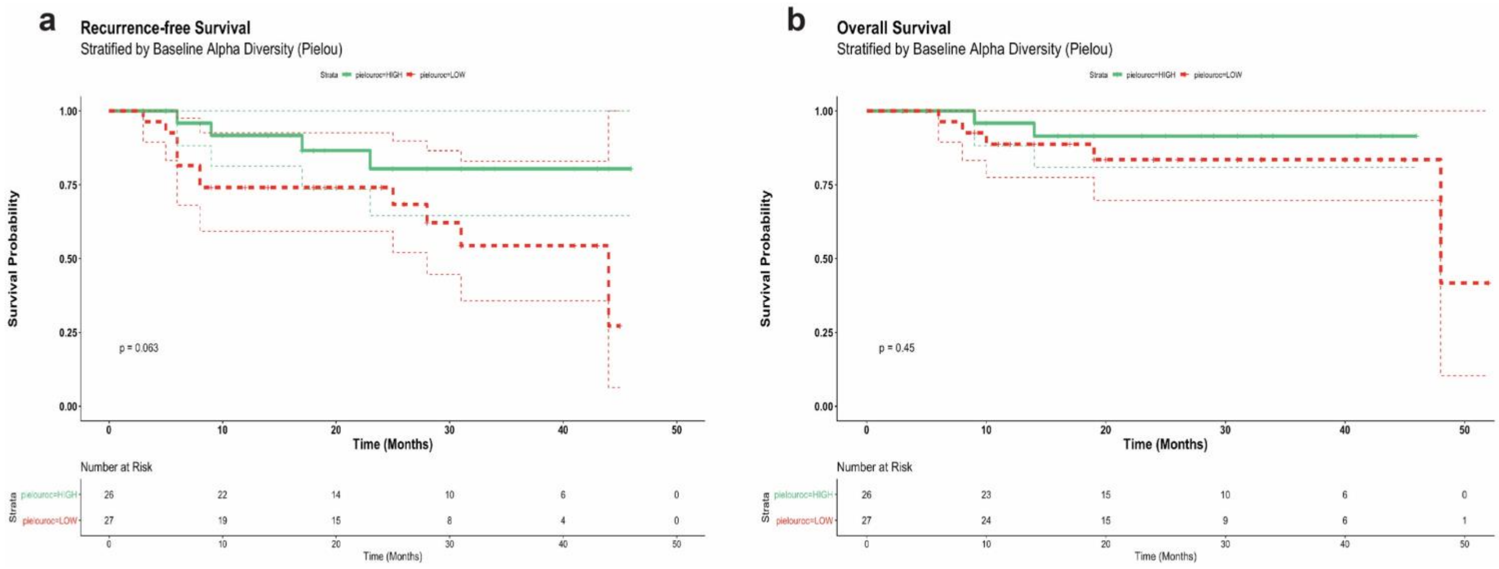

Supplemental Fig 3. Relationship between survival and alpha diversity (Pielou).

Kaplan-Meier curves for (A) recurrence free survival, (B) overall survival stratified by high and low alpha diversity (Pielou). Cases represent patients.

Supplemental Table 1. Patient and tumor characteristics (N=55)

	N	(%)
**Median age**, yrs (range)	48 (28-72)	—
**BMI**, Mean (SD), kg/m2	28.7(6.06)	—
**Race/Ethnicity**		
Asian	2	(3.6)
Black	4	(7.3)
Hispanic	24	(43.6)
White	24	(43.6)
Other	1	(1.8)
**FIGO Stage**		
IA1	1	(1.8)
IA2	0	(0)
IB1	5	(9.09)
IB2	6	(10.9)
IIA	3	(5.45)
IIB	28	(50.9)
IIIA	9	(16.3)
IIIB	0	(0)
IVA	3	(5.45)
IVB	0	(0)
**Grade**		
Well	4	(7.2)
Moderate	20	(36.3)
Poor	25	(45.4)
Unknown	6	(10.9)
**Histology**		
Squamous	43	(78.1)
Adenocarcinoma	8	(18.1)
Adenosquamous	3	(3.63)
**Node Level on PET**		
Common Iliac	9	(16.3)
External Iliac	23	(41.8)
Internal Iliac	5	(9.09)
Para-Aortic	3	(5.45)

None	15	(27.2)
**Median cervical tumor size (cm)**	5.4	—
**Smoking status**		
Current	4	(7.27)
Former	20	(36.3)
Never	31	(56.3)
**Antibiotic Use**		
No	14	(25.5)
Yes	41	(74.5)
**Brachytherapy**		
HDR	21	(38.2)
PDR	34	(61.8)
**Concurrent Chemotherapy (cycles)**		
Cisplatin		
(1-3)	2	(3.6)
(≥4)	51	(92.7)
Carboplatin		
(2)	1	(1.8)
Carboplatin + Cisplatin		
(2)+(2)	1	(1.8)

BMI, Body Mass Index; *FIGO*, International Federation of Gynecology and Obstetrics; *PET*, Pulsed Dose Rate; *HDR*, High Dose Rate ; *PDR*, Pulsed Dose Rate

Supplemental Table 2. Univariate Cox regression analysis for recurrence-free survival – Alpha Diversity all time points

**Characteristics**	**Univariate model**	
	**HR (95% CI)**	***P* value**
**Observed OTU**		
Baseline	0.99 (0.97-1)	0.21
Week 1	1 (0.97-1)	0.69
Week 3	0.99 (0.96-1)	0.59
Week 5	1 (0.98-1)	0.71
Week 12	1 (0.98-1)	0.77
**Shannon**		
Baseline	0.51 (0.23-1.1)	0.087
Week 1	0.94 (0.2-4.4)	0.94
Week 3	1.2 (0.25-5.6)	0.83
Week 5	0.83 (0.35-1.9)	0.66
Week 12	2.7 (0.13-57)	0.51
**Simpson**		
Baseline	0.025 (0.00036-1.7)	0.087
Week 1	13 (1.4e-05-1.2e+07)	0.13
Week 3	52 (6.6e-05-4.1e+07)	0.57
Week 5	0.31 (0.013-7.8)	0.48
Week 12	130000 (7.5e-13-2.2e+22)	0.56
**Inverse Simpson**		
Baseline	0.93 (0.84-1)	0.11
Week 1	0.96 (0.79-1.2)	0.69
Week 3	1 (0.95-1.1)	0.34
Week 5	1 (0.92-1.2)	0.54
Week 12	1.1 (0.81-1.4)	0.59
**Fisher**		
Baseline	0.95 (0.88-1)	0.23
Week 1	0.97 (0.86-1.1)	0.66
Week 3	0.96 (0.83-1.1)	0.6
Week 5	1 (0.91-1.2)	0.69
Week 12	1 (0.89-1.2)	0.81

CI, Confidence interval; *HR*, hazard ratio; *OTU*, operational taxonomic units.
∗Significant hazard ratios.
‡Significant *P* value <0.05.

Supplemental Table 3. Univariate Cox regression analysis for overall survival – Alpha Diversity all time points

Characteristics	Univariate model	
	HR (95% CI)	*P* value
**Observed OTU**		
Baseline	0.98 (0.95-1)	0.14
Week 1	0.98 (0.94-1)	0.35
Week 3	0.97 (0.92-1)	0.21
Week 5	1 (0.96-1)	0.98
Week 12	NA (NA-NA)	1
**Shannon**		
Baseline	0.34 (0.1-1.1)	0.08
Week 1	0.48 (0.063-3.7)	0.48
Week 3	1.2 (0.25-5.6)	0.83
Week 5	0.23 (0.037-1.4)	0.11
Week 12	NA (NA-NA)	1
**Simpson**		
Baseline	0.0059 (1.2e-05-2.9)	0.1
Week 1	0.45 (1.1e-08-1.8e+07)	0.93
Week 3	0.009 (1.7e-07-490)	0.4
Week 5	1.4 (0.00063-3200)	0.93
Week 12	NA (NA-NA)	1
**Inverse Simpson**		
Baseline	0.85 (0.7-1)	0.13
Week 1	0.86 (0.62-1.2)	0.39
Week 3	0.81 (0.61-1.1)	0.15
Week 5	0.89 (0.66-1.2)	0.46
Week 12	NA (NA-NA)	1
**Fisher**		
Baseline	0.91 (0.79-1)	0.15
Week 1	0.91 (0.74-1.1)	0.34
Week 3	0.84 (0.64-1.1)	0.22
Week 5	0.99 (0.79-1.3)	0.94
Week 12	NA (NA-NA)	1

CI, Confidence interval; *HR*, hazard ratio; *OTU*, operational taxonomic units.
∗Significant hazard ratios.
‡Significant *P* value <0.05.

Supplemental Table 4. Types of Antibiotics Taken by Study Participants During the Study Period

Type of antibiotic	Number of patients (N=41) N(%)
Trimethoprim / Sulfamethoxazole	19 (46.3)
Ciprofloxacin	10 (24.4)
Levofloxacin	4 (9.8)
Metronidazole	7 (17.1)
Amoxicillin / Clavulanic acid	3 (7.3)
Nitrofurantoin	2 (4.9)
Cefepime	2 (4.9)
Azithromycin	1 (2.4)
Bacitracin	1 (2.4)
Ampicillin / Sulbactam	1 (2.4)
Vancomycin	1 (2.4)
Cefpodoxime	1 (2.4)
Cefazolin	1 (2.4)
Amoxicillin	1 (2.4)
Piperacillin / Tazobactam	1 (2.4)
Vibramycin	1 (2.4)
