# Supplementary material for: Gut microbiome diversity is an independent predictor of survival in cervical cancer patients receiving chemoradiation
Source: Commun Biol. 2021 Feb 22;4:237. doi: 10.1038/s42003-021-01741-x (PMC7900251; doi:10.1038/s42003-021-01741-x)
Supplement: Supplementary file 3 — Description of Additional Supplementary Files [file 42003_2021_1741_MOESM3_ESM.pdf]

Description of Additional Supplementary Files

File name: Supplementary Data 1

Description: Data for Recreating KM Curves
