# Supplementary material for: Gut microbiome diversity is an independent predictor of survival in cervical cancer patients receiving chemoradiation
Source: Commun Biol. 2021 Feb 22;4:237. doi: 10.1038/s42003-021-01741-x (PMC7900251; doi:10.1038/s42003-021-01741-x)
Supplement: Supplementary file 5 — Reporting Summary [file 42003_2021_1741_MOESM5_ESM.pdf]

nature research

Corresponding author(s): Ann H. Klopp, MD, PhD
Manuscript ID: COMMSBIO-20-1670-T

Last updated by author(s): 12/16/2020

Reporting Summary

Nature Research wishes to improve the reproducibility of the work that we publish. This form provides structure for consistency and transparency in reporting. For further information on Nature Research policies, see our Editorial Policies and the Editorial Policy Checklist.

Statistics

For all statistical analyses, confirm that the following items are present in the figure legend, table legend, main text, or Methods section.

n/a	Confirmed	
☐	☒	The exact sample size (n) for each experimental group/condition, given as a discrete number and unit of measurement
☐	☒	A statement on whether measurements were taken from distinct samples or whether the same sample was measured repeatedly
☐	☒	The statistical test(s) used AND whether they are one- or two-sided<br>*Only common tests should be described solely by name; describe more complex techniques in the Methods section.*
☐	☒	A description of all covariates tested
☐	☒	A description of any assumptions or corrections, such as tests of normality and adjustment for multiple comparisons
☐	☒	A full description of the statistical parameters including central tendency (e.g. means) or other basic estimates (e.g. regression coefficient) AND variation (e.g. standard deviation) or associated estimates of uncertainty (e.g. confidence intervals)
☐	☒	For null hypothesis testing, the test statistic (e.g. F, t, r) with confidence intervals, effect sizes, degrees of freedom and P value noted<br>*Give P values as exact values whenever suitable.*
☒	☐	For Bayesian analysis, information on the choice of priors and Markov chain Monte Carlo settings
☒	☐	For hierarchical and complex designs, identification of the appropriate level for tests and full reporting of outcomes
☒	☐	Estimates of effect sizes (e.g. Cohen's d, Pearson's r), indicating how they were calculated

Our web collection on statistics for biologists contains articles on many of the points above.

Software and code

Policy information about availability of computer code

Data collection	N/A
Data analysis	RStudio Version 1.3.1093

For manuscripts utilizing custom algorithms or software that are central to the research but not yet described in published literature, software must be made available to editors and reviewers. We strongly encourage code deposition in a community repository (e.g. GitHub). See the Nature Research guidelines for submitting code & software for further information.

Data

Policy information about availability of data

All manuscripts must include a data availability statement. This statement should provide the following information, where applicable:

- Accession codes, unique identifiers, or web links for publicly available datasets
- A list of figures that have associated raw data
- A description of any restrictions on data availability

The sequencing datasets generated during and/or analysed during the current study are available in the Sequence Read Archive (SRA) repository (accession number: PRJNA685389). All data generated or analysed during this study are included in this published article (and its supplementary information files)

Field-specific reporting

Please select the one below that is the best fit for your research. If you are not sure, read the appropriate sections before making your selection.

☒ Life sciences ☐ Behavioural & social sciences ☐ Ecological, evolutionary & environmental sciences

For a reference copy of the document with all sections, see nature.com/documents/nr-reporting-summary-flat.pdf

Life sciences study design

All studies must disclose on these points even when the disclosure is negative.

Sample size	No sample size calculation was performed. Gut microbiome and cervical swab samples were collected prospectively from cervical cancer patients according to a protocol approved by The University of Texas MD Anderson Cancer Center Institutional Review Board (MDACC 2014-0543) for patients with biopsy-proven carcinoma of the cervix treated at MD Anderson and the Lyndon B. Johnson Hospital Oncology Clinic from September 22, 2015, to January 11, 2019.
Data exclusions	No data were excluded from the analysis
Replication	Our group previously compared data from 16s rRNA and Whole Genome Sequencing (WGS. We found that diversity, evenness, and richness measures are comparable when using 16s rRNA and WGS, while relative abundances of individual genera differed between the two methods.
Randomization	n/a
Blinding	n/a, this is a prospective observational study.

Reporting for specific materials, systems and methods

We require information from authors about some types of materials, experimental systems and methods used in many studies. Here, indicate whether each material, system or method listed is relevant to your study. If you are not sure if a list item applies to your research, read the appropriate section before selecting a response.

Materials & experimental systems

n/a	Involved in the study	
☒	☐	Antibodies
☒	☐	Eukaryotic cell lines
☒	☐	Palaeontology and archaeology
☒	☐	Animals and other organisms
☐	☒	Human research participants
☐	☒	Clinical data
☒	☐	Dual use research of concern

Methods

n/a	Involved in the study	
☒	☐	ChIP-seq
☒	☐	Flow cytometry
☒	☐	MRI-based neuroimaging

Human research participants

Policy information about studies involving human research participants

Population characteristics	Inclusion criteria included all patients who had new a diagnoses of locally advanced, nonmetastatic carcinoma of the cervix and underwent definitive CRT with EBRT followed by brachytherapy. None of the patients included in this cohort underwent surgery. Ineligibility criteria included currently pregnant or lactating women. Patients received a minimum of 45 Gy via EBRT in 25 fractions over 5 weeks with weekly cisplatin followed by two brachytherapy sessions at approximately weeks 5 and 7 with EBRT in between for gross nodal disease or persistent disease in the parametrium. Patients with stage IB1 cancer were given CRT due to the presence of nodal disease. Additionally, details such as place of residence, lifestyle and diet were not known in our data set. Furthermore, we acknowledged the observation that factors such as racial variation have been shown to contribute to differences in host genetics and innate/adaptive immunity and could have impacted our overall outcomes.
Recruitment	Gut microbiome and cervical swab samples were collected prospectively from cervical cancer patients according to a protocol approved by The University of Texas MD Anderson Cancer Center Institutional Review Board (MDACC 2014-0543) for patients with biopsy-proven carcinoma of the cervix treated at MD Anderson and the Lyndon B. Johnson Hospital Oncology Clinic from September 22, 2015, to January 11, 2019
Ethics oversight	The study protocol was approved The University of Texas MD Anderson Cancer Center Institutional Review Board (MDACC 2014-0543). Written informed consent from eligible patients willing to participate in this study was obtained.

Note that full information on the approval of the study protocol must also be provided in the manuscript.

Clinical data

Policy information about clinical studies

All manuscripts should comply with the ICMJE guidelines for publication of clinical research and a completed CONSORT checklist must be included with all submissions.

Clinical trial registration	n/a
Study protocol	(MDACC 2014-0543)
Data collection	Gut microbiome and cervical swab samples were collected prospectively from cervical cancer patients according to a protocol approved by The University of Texas MD Anderson Cancer Center Institutional Review Board (MDACC 2014-0543) for patients with biopsy-proven carcinoma of the cervix treated at MD Anderson and the Lyndon B. Johnson Hospital Oncology Clinic from September 22, 2015, to January 11, 2019.
Outcomes	For microbiome analysis, rarefaction depth was set at 7066 reads. The Shannon diversity index (SDI) was used to evaluate α-diversity (within samples), and principle coordinates analysis of unweighted UniFrac distances was used to examine β-diversity (between samples). A receiver operating characteristic (ROC) curve was calculated using a linear regression model. A table was constructed using the coordinates of the curve in order to identify the most appropriate cut-off for SDI. The cut-off for SDI was identified to be 2.69. Patient and tumor characteristics were analyzed by univariate and multivariate Cox regression models for Recurrence-free survival (RFS) and Overall survival (OS). Covariates with a p-value of less than or equal to 0.1 in the univariate model were chosen to be included in the multivariate analysis. Recurrence free survival (RFS) was defined as the time from the initiation of the therapy until the date of disease relapse or progression or death from any cause. Disease relapse or progression was defined as appearance or signs of the disease that was confirmed pathologically or radiographically. Overall survival (OS) is defined as the time from the date of initiation of therapy until the date of death from any cause. Survival was estimated by the Kaplan–Meier method, and comparisons were made by log-rank tests. Characteristics included age, body mass index (BMI), race, FIGO stage, grade, histology, nodal status, smoking status, antibiotic use and max tumor size. For each outcome of interest, a multivariate Cox regression analysis was performed to adjust for the effects of prognostic factors identified on univariate analysis as influencing survival in cervical cancer. These analyses were conducted using covariates with $p \leq 0.1$ in a stepwise fashion. We also ran a correlation analysis of alpha diversity metrics with tumor flow cytometry markers using a linear regression and Spearman's correlation. Alpha (within sample) diversity was evaluated using SDI. The relative abundance of microbial taxa, classes, and genera present in long term vs short term survivors was determined using LDA Effect Size54, applying the one-against-all strategy with a threshold of 3.5 for the logarithmic LDA score for discriminative features and α of 0.05 for factorial Kruskal-Wallis testing among classes. Long term survivors were classified as patients who had a follow up of two years or more and were alive at time of last follow up, while short term survivors had a follow up of one year or less. LDA Effect Size analysis was restricted to bacteria present in 20% or more of the study population. Kaplan-Meier curves were generated for patients with normal BMI and overweight/obese BMI based on Cox analysis and clostridia abundance (BMI cut-offs were based on WHO standards). The significance of differences was determined using the log-rank test. Statistical significance was set at an alpha of 5% for a two-sided p-value. Analyses were conducted using Rstudio version Orange Blossom – 1.2.5033.
